# THE HEALTH-RELATED TASKS PAID CAREGIVERS IN NEW YORK STATE PERFORM IN THE HOME

**DOI:** 10.1093/geroni/igz038.790

**Published:** 2019-11-08

**Authors:** Jennifer M Reckrey, Emma Tsui, R S Morrison, Emma Geduldig, Robyn Stone, Katherine Ornstein, Alex Federman

**Affiliations:** 1 Icahn School of Medicine at Mount Sinai, New York, New York, United States; 2 CUNY Graduate School of Public Health and Health Policy, New York, New York, United States; 3 Leading Age, Washington, District of Columbia, United States

## Abstract

Paid caregivers (e.g. home health aides, personal care attendants) are formally tasked with helping older adults with functional impairment meet their basic needs at home. This study used semi-structured interviews (n=30) with dyads of patients or their proxies and their paid caregivers in New York City to 1) understand the range of health-related tasks paid caregivers perform in the home and 2) determine if these tasks are taught in the New York State government’s Department of Health curricula. We found that patients, proxies, and paid caregivers all described that paid caregivers performed a wide range of health-related tasks that were often not a part of their formal training. Creating clear competencies for paid caregivers that reflect the full breadth of health-related tasks they may perform at home will help maximize the potentially positive impact of the paid caregiver workforce on the lives of patients living at home with functional impairment.

